# Identifying Subgroups among Hardcore Smokers: a Latent Profile Approach

**DOI:** 10.1371/journal.pone.0133570

**Published:** 2015-07-24

**Authors:** Jeroen Bommelé, Marloes Kleinjan, Tim M. Schoenmakers, William J. Burk, Regina van den Eijnden, Dike van de Mheen

**Affiliations:** 1 IVO Addiction Research Institute, Rotterdam, The Netherlands; 2 Erasmus Medical Centre, Rotterdam, The Netherlands; 3 Trimbos Institute (Netherlands Institute of Mental Health and Addiction), Utrecht, The Netherlands; 4 Behavioural Science Institute, Radboud University Nijmegen, Nijmegen, The Netherlands; 5 Faculty of Social Sciences, Utrecht University, Utrecht, The Netherlands; 6 Department of Health Promotion, Maastricht University, Maastricht, The Netherlands; Legacy, Schroeder Institute for Tobacco Research and Policy Studies, UNITED STATES

## Abstract

**Introduction:**

Hardcore smokers are smokers who have little to no intention to quit. Previous research suggests that there are distinct subgroups among hardcore smokers and that these subgroups vary in the perceived pros and cons of smoking and quitting. Identifying these subgroups could help to develop individualized messages for the group of hardcore smokers. In this study we therefore used the perceived pros and cons of smoking and quitting to identify profiles among hardcore smokers.

**Methods:**

A sample of 510 hardcore smokers completed an online survey on the perceived pros and cons of smoking and quitting. We used these perceived pros and cons in a latent profile analysis to identify possible subgroups among hardcore smokers. To validate the profiles identified among hardcore smokers, we analysed data from a sample of 338 non-hardcore smokers in a similar way.

**Results:**

We found three profiles among hardcore smokers. ‘Receptive’ hardcore smokers (36%) perceived many cons of smoking and many pros of quitting. ‘Ambivalent’ hardcore smokers (59%) were rather undecided towards quitting. ‘Resistant’ hardcore smokers (5%) saw few cons of smoking and few pros of quitting. Among non-hardcore smokers, we found similar groups of ‘receptive’ smokers (30%) and ‘ambivalent’ smokers (54%). However, a third group consisted of ‘disengaged’ smokers (16%), who saw few pros and cons of both smoking and quitting.

**Discussion:**

Among hardcore smokers, we found three distinct profiles based on perceived pros and cons of smoking. This indicates that hardcore smokers are not a homogenous group. Each profile might require a different tobacco control approach. Our findings may help to develop individualized tobacco control messages for the particularly hard-to-reach group of hardcore smokers.

## Introduction

Smoking is one of the leading causes of preventable death in the world. Reducing its prevalence would improve health globally [1]. An important predictor of quitting attempts is motivation to quit smoking [2]. We therefore need to investigate ways of increasing motivation to quit smoking, especially among smokers with no or low intention to quit.

Hardcore smokers are a group of smokers who have little to no intention to quit. In general, they also smoke heavily and have been smoking for a considerable number of years [3]. Previous research indicated that hardcore smokers are less affected by current tobacco control policies than non-hardcore smokers [4–6]. To reach hardcore smokers and motivate them to quit, we require specialized interventions [4,6]. These interventions should ideally contain individualized tobacco control messages (i.e. tailored information) based on individual characteristics [7].

Some studies suggest that distinct subgroups (‘profiles’) exist among smokers with low intention to quit [8–11]. Dijkstra and De Vries [12], for example, distinguished five profiles among so-called ‘pre-contemplators’ [13]. While pre-contemplators do not intend to quit smoking within 6 months, they could be occasional or light smokers. Hardcore smokers also do not intend to quit within 6 months, but they smoke at least 15 cigarettes per day and have been smoking for many years. Given that there is heterogeneity in pre-contemplators, one might also expect different profiles among hardcore smokers. Identifying such profiles could help to develop interventions using individualized health promoting messages for hardcore smokers. This could improve the smoking cessation interventions for this group.

According to stage models, such as the Transtheoretical Model, perceived pros and cons indicate motivation to quit, which would predict smoking cessation [13,14]. The profiles found among pre-contemplators varied, besides quitting self-efficacy, in the number of pros and cons of quitting [12]. Among pre-contemplators, Dijkstra and De Vries [12] distinguished between *motivated* smokers, who have many pros of quitting and few cons of quitting; *disengaged* smokers, who scored below average on both pros and cons of quitting; and *unmotivated* smokers, who have few pros of quitting and many cons of quitting. Others also found three similar groups in pre-contemplators [8]. Based on this, we expected to find comparable profiles in our sample of hardcore smokers. As profiles among pre-contemplators vary in their perceived pros and cons, profiles among hardcore smokers may therefore also vary with regard to the perceived pros and cons.

In a previous study, we qualitatively examined perceived pros and cons of smoking and quitting among hardcore smokers [15]. In that study, we found that perceived pros and cons of smoking differed from those of quitting. Weight gain, for example, is an important con of quitting, but weight maintenance was not an important pro of smoking. Also, many believed smoking helped them to maintain social contacts, but few believed they would lose friends if they quit smoking. We therefore concluded that both the pros and cons of both smoking and quitting seem theoretically relevant for identifying profiles among hardcore smokers.

In the current study, we used the perceived pros and cons of both smoking and quitting to identify distinct profiles among hardcore smokers. We compared these profiles on quitting self-efficacy, nicotine dependence and smoking history. These covariates are relevant, because hardcore smokers tend to have a lower quitting self-efficacy [6], have higher nicotine dependence [16], and started smoking earlier in life than non-hardcore smokers [4].

A first profile could include motivated smokers who see many cons of smoking and many pros of quitting. They may know that quitting would be beneficial, but may be unable to quit due to their high levels of nicotine dependence [2]. We expected a second profile to include smokers who are rather neutral towards the pros and cons of smoking and quitting. These hardcore smokers may be less nicotine dependent and would experience fewer smoking-related problems, such as withdrawal symptoms [17] or nocturnal craving [18]. They may be less motivated to quit, because they have not yet explicitly considered the benefits of quitting. Finally, we expected a third profile whose members perceived many pros of smoking, but few pros of quitting. Like the unmotivated pre-contemplators in Dijkstra and De Vries [12], they are probably unmotivated to quit smoking; thinking about quitting may be too threatening for them [19] or perhaps they genuinely do not care about quitting. In practice, this profile may be especially hard to reach through current tobacco-control efforts.

In addition to identifying different profiles, we also investigated which profiles are unique to hardcore smokers. Hardcore and non-hardcore smokers differ in their beliefs about smoking. Hardcore smokers are, for example, less likely to acknowledge the dangers of smoking to their own health or to the health of others [4]. Profiles among hardcore smokers may therefore be different from those among non-hardcore smokers. To investigate such differences, we included a separate sample of non-hardcore smokers who had no intention to quit within six months (i.e., non-hardcore pre-contemplators). This sample is similar to the ones in Dijkstra and De Vries [12] and in similar studies on smokers with low intentions to quit [4,20].

Non-hardcore smokers are generally more positive towards quitting than hardcore smokers [4,5]. Among non-hardcore smokers, we therefore expected to find at least one profile of receptive, but more nicotine dependent non-hardcore smokers. As hardcore and non-hardcore smokers also differ in other beliefs, any additional profile found among non-hardcore smokers may differ from those found among hardcore smokers.

In summary, in this study we used the perceived pros and cons of smoking and quitting to identify profiles among hardcore smokers. We then compared these profiles, using relevant smoking-related variables, such as quitting self-efficacy, nicotine dependence and smoking history. Finally, to investigate how unique they are, we compared them with profiles from a sample of non-hardcore smokers.

## Material and Methods

### Procedure

Respondents were recruited via an online survey sample (Survey Sampling International). Survey Sampling International has about 11.5 million panellist in 103 countries. From July 2012 to September 2012, Dutch panel members filled out a small selection screener questionnaire that contained the criteria below. We identified 542 hardcore smokers and 367 non-hardcore smokers, and invited all to complete our online survey. To obtain a stratified sample, we pursued an equal representation of sex and socioeconomic status (SES). We distinguished two SES groups, based on participants’ highest completed level of education (Dutch abbreviations in brackets). Low SES had primary education, lower secondary education (MAVO), or lower to middle level vocational education (LBO, MBO). High SES had higher secondary education (HAVO, VWO) or tertiary education (HBO, University).

We defined ‘hardcore’ smokers as those who a) were aged 35 or older, b) smoked every day, c) smoked on average 15 cigarettes or more a day, d) had not attempted to quit smoking in the past year, e) had smoked at least 15 years in their lifetime, and f) had no intention to quit within 6 months. Non-hardcore smokers were those who a) were aged 35 or older, b) smoked ‘every day’ or ‘sometimes’, c) had no intention to quit within 6 months, and d) did not meet all criteria for hardcore smokers.

We excluded a small number of participants who showed an obvious lack of motivation to complete the survey honestly. They either answered all items within a scale identically (i.e. straight-lining, n = 55) or gave obvious counterfactual statements about their smoking or quitting history (n = 14). Some did both (n = 2). The remaining 510 hardcore and 338 non-hardcore smokers were included in the analyses.

### Measures

#### Demographics and smoking characteristics

We obtained both basic demographics (i.e. sex, age and SES) and smoking-related characteristics (i.e. age of onset, years smoked in life and intention to quit). Years smoked in life and intention to quit were screener variables we used to identify eligible participants.

#### Nicotine dependence

To measure nicotine dependence, we used the Dutch version of the Fagerström Test for Nicotine Dependence [21,22]. This six item scale assesses the number of cigarettes smoked per day, time to first cigarette after awakening, and difficulty to refrain from smoking in certain situations. The Fagerström questionnaire includes a categorical item to measure cigarettes per day (i.e. 10 or less; 11–20; 21–30; and 31 or more). We used a separate continuous item to measure cigarettes per day more precisely for the demographic measures.

#### Quitting self-efficacy

We used a 16-item self-efficacy scale (α = .95) to measure the perceived ability to maintain abstinent after a hypothetical quitting attempt [23]. Each question began with ‘Imagine you have quit smoking. Would you be able to refrain from smoking when…?’ Respondents then indicated their perceived ability in various situations, such as ‘being with friends’, ‘feeling angry’, and ‘craving for cigarettes’. The response options ranged from *absolutely not* (1) to *most certainly* (7). This self-efficacy measure has proven reliable in smokers with low quitting intentions [12,24].

#### Pros and cons of smoking and quitting

We used four separate scales to measure the perceived pros of smoking, cons of smoking, pros of quitting and cons of quitting. Each scale had 16 statements and participants indicated their level of agreement with each statement. The endpoints were labelled *strongly disagree* (1) to *strongly agree* (7). The topics of these scales where money, health, intrapersonal processes (e.g., stress), social environment, physical environment (e.g. smell of cigarettes) and weight gain. All items were based on a focus group study conducted among another sample of hardcore smokers [15]. Example items were ‘Smoking helps me fight boredom’ (pro of smoking); ‘I feel addicted to smoking’ (con of smoking); ‘Quitting would improve my health’ (pro of quitting); and ‘Quitting would make me gain weight’ (con of quitting). To avoid order effects, we counterbalanced the four scales. We calculated the Cronbach’s alpha for the pros of smoking scale (α = .81), the cons of smoking scale (α = .85), the pros of quitting scale (α = .89) and the cons of quitting scale (α = .79). Reliability was acceptable for all scales.

### Statistical analysis

To identify profiles among both hardcore and non-hardcore smokers, we conducted two separate series of latent profile analyses (LPA) in MPlus [25], in which we included the pros and cons of smoking and quitting scales as predictors. A latent profile analysis is a person-oriented approach to identify distinct, homogeneous subgroups. These subgroups are referred to as latent profiles or classes [26]. We performed a series of models, with each specifying between one and six classes. Theoretical and statistical considerations (i.e., goodness-of-fit indices) were used to identify the most parsimonious number of profiles that appropriately fit the observed data [26]. To identify the optimal number of profiles in all analyses, we primarily used the Bootstrap Likelihood Ratio Test (BLRT) [27]. The BLRT compares a solution specifying a certain number of profiles (e.g., three profiles) with a solution specifying one fewer profiles (i.e. two profiles). Significant *p*-values indicate the profile solution with the higher number of class better fits the data. A non-significant *p*-values indicate an equivalent fit between two solutions, with the more parsimonious solution then being preferred. In addition to the BLRT, we also considered other statistical indicators, such as the Akaike Information Criteria (AIC) [28], Bayesian Information Criterion (BIC) [29] and entropy. Entropy is an index ranging from 0 to 1 that indicates how accurate participants are classified in their profiles, with a higher value suggesting a better fit (cf. [30]).

After we performed the LPAs, we used IBM SPSS Statistics 19 to compare all profiles on variables not included in the latent profile analyses (i.e., demographics, smoking history, nicotine dependence, and quitting self-efficacy). We used ANOVA’s to compare profiles on continuous variables and chi-square analyses to compare profiles on nominal and ordinal variables. To account for uncertainty of profile membership in these analyses, we used the posterior probabilities of profile membership as weights (cf. [31,32]).

All data used in this study are publicly available from the Open Science Framework (https://osf.io/5brnq/).

### Ethics statement

The Medical Ethical Committee of the Erasmus MC declared that the Medical Research Involving Human Subjects Act (also known by its Dutch abbreviation WMO) does not apply to this study. It had therefore no objection to the execution of this research. None of the authors had access to identifying participant information at any time.

## Results

### Sample characteristics

#### Demographics and smoking characteristics

Table 1 shows the background characteristics. Hardcore smokers were older than non-hardcore smokers, *F*(1, 846) = 4.653, *p* = .031, η^2^ = .005. They also started smoking at younger age, *F*(1, 846) = 5.359, *p* = .021, η^2^ = .006, had smoked more years in life, *F*(1, 846) = 42.338, *p* < .001, η^2^ = .048, had higher nicotine dependence scores, *F*(1, 846) = 226.024, *p* < .001, η^2^ = .211, smoked more cigarettes per day, *F*(1, 846) = 376.353, *p* < .001, η^2^ = .308 and had higher intention to quit, *χ* ^*2*^ (3, N = 848) = 25.744, *p* < .001, *φ* = .174. We found no differences on sex, *χ* ^*2*^ (1, N = 848) = 2.763, *p* = .096, *φ* = .057 and SES, *χ* ^*2*^ (1, N = 848) = .249, *p* = .618, *φ* = .017.

**Table 1:** Sample characteristics.

	Hardcore smokers (n = 510)	Non-hardcore smokers (n = 338)	*Significance*
*Demographics*			
Female, n (%)	50.4	56.2	NS
Age (*SD*)	52.7 (7.2)	51.6 (7.7)	*p* = .031
*Socioeconomic status (%)* ^a^			
Low	56.5	54.7	NS
High	43.5	45.3	
*Smoking History*			
Age of smoking onset (*SD*)	16.3 (5.5)	17.2 (4.9)	*p* = .021
Years smoked in life (*SD*) ^b^	35.4 (8.4)	31.2 (10.2)	*p* < .001
*Nicotine dependence*			
FTND (*SD*)	5.3 (1.8)	3.1 (2.4)	*p* < .001
Cigarettes per day (*SD*)	21.2 (6.6)	11.1 (8.4)	*p* < .001
*Intention to quit (%)*			
Within 1 year	11.6	23.4	*p* < .001
Within 5 years	18.0	13.0	
Not quitting, but smoking less	32.4	34.6	
Not quitting, not smoking less	38.0	29.0	
*Smoking-related beliefs*			
Quitting self-efficacy (SD) ^c^	3.8 (1.1)	4.2 (1.0)	*p* < .001
Pros of smoking (*SD*)	3.5 (0.7)	3.3 (0.7)	*p* < .001
Cons of smoking (*SD*)	4.5 (0.8)	4.4 (0.8)	*p* = .026
Pros of quitting (*SD*)	4.5 (0.8)	4.5 (0.9)	NS
Cons of quitting (*SD*)	3.5 (0.7)	3.3 (0.7)	*p* < .001

^a^ Socioeconomic status was measured as the highest completed education.

^b^ Hardcore smokers had smoked > 15 years by definition.

^c^ Higher scores indicate more quitting self-efficacy.

#### Quitting self-efficacy

Hardcore smokers had lower quitting self-efficacy scores than non-hardcore smokers, *F*(1, 846) = 32.187, *p* < .001, η^2^ = .037.

#### Pros and cons of smoking and quitting

Hardcore smokers had higher scores on the pros of smoking, *F*(1, 846) = 18.203, *p* < .001, η^2^ = .021, the cons of smoking, *F*(1, 846) = 4.994, *p* = .026, η^2^ = .006, and the cons of quitting, *F*(1, 846) = 21.038, *p* < .001, η^2^ = .024, than non-hardcore smokers. We found no significant difference in pros of quitting, *F*(1, 846) = .230, *p* = .631, η^2^ < .001.

### Latent profile analyses

We analysed the sample of hardcore smokers and the sample of non-hardcore smokers separately. Table 2 shows the goodness-of-fit indices (AIC, BIC, entropy, and BLRT) for the series of LPAs of 510 hardcore smokers. Based on these goodness-of-fit indices, the most parsimonious solution included three profiles. We labelled each profile according to characteristics of its members. Table 3 shows the background characteristics of all profiles.

**Table 2:** Latent profile analysis models in hardcore smokers and non-hardcore smokers.

	AIC	BIC	BIC (Adjusted)	Entropy	BLRT H0 LL-value	*p*-value
*Hardcore smokers*						
1 Class	4552.599	4586.474	4561.081	-	-	-
2 Classes	4309.581	4364.628	4323.364	.618	-2268.299	.0401
**3 Classes**	**4167.676**	**4243.896**	**4186.761**	**.775**	**-2141.790**	**.0439**
4 Classes	4099.934	4197.325	4124.320	.773	-2065.838	.0870
5 Classes	4041.397	4159.961	1071.085	.750	-2026.967	.2328
*Non-hardcore smokers*						
1 Class	3181.690	3212.275	3186.898	-	-	-
2 Classes	3002.840	3052.540	3011.302	.694	-1582.845	.0039
**3 Classes**	**2879.252**	**2948.067**	**2890.968**	**.806**	**-1488.420**	**.0019**
4 Classes	2839.478	2927.408	2854.449	.795	-1421.626	.1420
5 Classes	2800.667	2907.712	2818.892	.810	-1396.739	.0436

*Note*: The optimal number of classes is presented in bold.

**Table 3:** Characteristics of profiles in hardcore smokers and non-hardcore smokers.

	Hardcore smokers (N = 510)			Non-hardcore smokers (N = 338)		
	Receptive (n = 186)	Ambivalent (n = 300)	Resistant (n = 24)	Receptive (n = 101)	Ambivalent (n = 184)	Disengaged (n = 53)
*Demographics*						
Female, N (%)	53.8 ^a^	48.0 ^a^	54.2 ^a^	59.4 ^a^	53.8 ^a^	58.5 ^a^
Age (*SD*)	52.3 (7.3) ^a^	52.9 (7.2) ^a^	54.3 (6.5) ^a^	51.3 (6.9) ^a^	51.2 (8.1) ^a^	53.9 (7.6) ^a^
*Socioeconomic status*						
Low (%)	51.6 ^a^	59.7 ^a^	54.2 ^a^	59.4 ^a^	53.3 ^a^	50.9 ^a^
High (%)	48.4 ^a^	40.3 ^a^	45.8 ^a^	40.6 ^a^	46.7 ^a^	49.1 ^a^
*Smoking history*						
Age of smoking onset (*SD*)	16.3 (4.1) ^a^	16.3 (5.6) ^a^	16.2 (11.0) ^a^	16.2 (3.8) ^a^	17.7 (5.4) ^b^	17.3 (4.9) ^ab^
Years smoked in life (*SD*)	34.9 (8.5) ^a^	35.5 (8.2) ^a^	37.0 (9.8) ^a^	31.4 (9.1) ^a^	30.9 (10.4) ^a^	31.8 (11.3) ^a^
Nicotine dependence						
FTND (*SD*)	5.54 (1.81) ^a^	5.09 (1.77) ^b^	5.54 (2.02) ^ab^	3.98 (2.37) ^a^	3.02 (2.23) ^b^	1.85(2.14) ^c^
Cigarettes per day (*SD*)	22.0 (6.6) ^a^	20.8 (6.6) ^a^	19.9 (6.4) ^a^	13.3 (11.0) ^a^	11.1 (7.2) ^b^	7.1 (4.4) ^c^
*Intention to quit (%)*						
Within 1 year	21.5 ^a^	6.3 ^b^	0 ^b^	38.6 ^a^	19.0 ^b^	9.4 ^c^
Within 5 years	25.8 ^a^	14.0 ^b^	8.3 ^b^	19.8 ^a^	11.4 ^b^	5.7 ^c^
Not quitting, but smoking less	31.2 ^a^	34.0 ^b^	20.8 ^b^	28.7 ^a^	38.6 ^b^	32.1 ^c^
Not quitting, not smoking less	21.5 ^a^	45.7 ^b^	70.8 ^b^	12.9 ^a^	31.0 ^b^	52.8 ^c^
*Smoking-related beliefs*						
Quitting self-efficacy (*SD*)	3.85 (1.07) ^a^	3.80 (1.06) ^a^	3.63 (1.65) ^a^	4.06 (.91) ^a^	4.16 (.85) ^a^	4.78 (1.27) ^b^
Pros of smoking (*SD*)	3.09 (.57) ^a^	3.75 (.58) ^b^	4.41 (.77) ^c^	3.12 (.73) ^a^	3.44 (.64) ^b^	3.37 (.87) ^b^
Cons of smoking (*SD*)	5.16 (.45) ^a^	4.20 (.45) ^b^	2.70 (.42) ^c^	5.25 (.44) ^a^	4.24 (.42) ^b^	3.04 (.45) ^c^
Pros of quitting (*SD*)	5.25 (.58) ^a^	4.26 (.58) ^b^	2.74 (.64) ^c^	5.49 (.47) ^a^	4.36 (.43) ^b^	3.23 (.50) ^c^
Cons of quitting (*SD*)	3.29 (.61) ^a^	3.71 (.62) ^b^	3.62 (1.03) ^b^	3.23 (.72) ^a^	3.47 (.62) ^b^	3.07 (.87) ^a^

*Note*: Profiles were based on pros and cons scales. If profiles within a sample share a superscript character, they are not significantly different from each other (*p* < .05).

**Fig 1:**
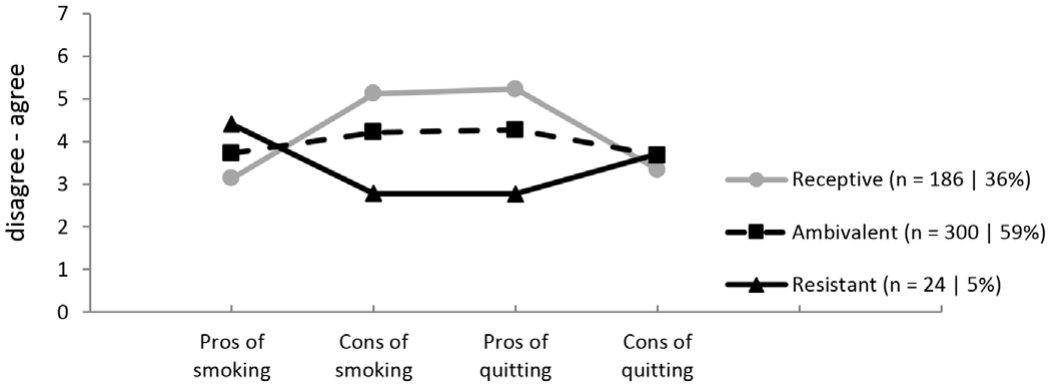
Mean scores on pros and cons of smoking and quitting in hardcore smokers. Higher scores indicate higher average agreement with pros or cons.

Among hardcore smokers, the first profile (36%) was labelled *‘receptive’*. Compared to members of other profiles, receptive hardcore smokers scored lower on the pros of smoking and the cons of quitting, and higher on the cons of smoking and pros of quitting. The second profile (59%) was labelled ‘*ambivalent’* and included smokers who scored around the neutral point (4) on all four scales. The third and final profile (5%) was labelled ‘*resistant’*. Compared to members of other profiles, *resistant* hardcore smokers scored higher on the pros of smoking, but lower on the cons of smoking and the pros of quitting than members of other profiles. Fig 1 shows average pros and cons scores for these three profiles.

The LPA for non-hardcore smokers indicated that the most parsimonious solution also included three profiles (Table 2). As two profiles among non-hardcore smokers were similar to those found among hardcore smokers, they were labelled similarly. The first profile among non-hardcore smokers (30%) was labelled ‘*receptive’*. Its members scored higher on the cons of smoking and the pros of quitting than members of the other two profiles. The second and largest profile (54%) was labelled ‘*ambivalent’* and included members who scored around the neutral point (4) on all four measures. The third profile (16%) included members who disagreed with all four scales. This suggests that members of this profile are psychologically uninvolved in both smoking and quitting. We therefore labelled this profile ‘*disengaged’*.

### Profile characteristics of hardcore smokers

#### Demographics and smoking characteristics

Among hardcore smokers, we found no significant differences in age, *F*(2, 506) = .992, *p* = .371, η^2^ = .004, sex, *χ* ^*2*^ (2, N = 510) = 1.669, *p* = .434, *φ* = .057, SES, *χ* ^*2*^ (2, N = 510) = 3.084, *p* = .214, *φ* = .078, age of onset, *F*(2, 506) = .006, *p* = .994, η^2^ < .001, and years smoked in life, *F*(2, 506) = .745, *p* = .475, η^2^ = .003. We found a significant difference between groups in intention to quit, *χ* ^*2*^ (6, N = 510) = 62.002, *p* < .001, *φ* = .349.

#### Nicotine dependence

We found one single significant difference in FTND scores, *F*(2, 506) = 3.848, *p* = .022, η^2^ = .015. Receptive hardcore smokers were more nicotine dependent than ambivalent hardcore smokers, *p* = .008. We found no other difference in nicotine dependence. We found also no difference between profiles in cigarettes per day, *F*(2,506) = 2.523, *p* = .081, η^2^ = .010.

#### Quitting self-efficacy

We found no differences between profiles in quitting self-efficacy, *F*(2,506) = .455, *p* = .634, η^2^ = .002.

### Profile characteristics of non-hardcore smokers

#### Demographics and smoking characteristics

Among non-hardcore smokers, we found no significant differences in age, *F*(2, 334) = 2.652, *p* = .072, η^2^ = .016, sex, *χ* ^*2*^ (2, N = 338) = 964, *p* = .618, *φ* = .053, and SES, *χ* ^*2*^ (2, N = 338) = 1.358, *p* = .507, *φ* = .063. Age of onset differed between profiles, *F*(2, 334) = 3.076, *p* = .047, η^2^ = .018. Post hoc analyses showed that receptive non-hardcore smokers began smoking earlier in life than ambivalent non-hardcore smokers, *p* = .014. We found no other significant difference in age of onset. We also found no difference in years smoked in life, *F*(2,334) = .191, *p* = .827, η^2^ = .001. Intention to quit was different between groups, *χ* ^*2*^ (6, N = 338) = 43.717, *p* < .001, *φ* = .360.

#### Nicotine dependence

All three profiles differed significantly in FTND scores, *F*(2, 334) = 15.856, *p* < .001, η^2^ = .087. Receptive non-hardcore smokers were more nicotine dependent than both ambivalent non-hardcore smokers, *p* = .001, and disengaged non-hardcore smokers, *p* < .001. Ambivalent non-hardcore smokers were more nicotine dependent than disengaged non-hardcore smokers, *p* = .001. All three profiles also differed in the number of cigarettes per day, *F*(2, 334) = 9.788, *p* < .001, η^2^ = .055. Receptive non-hardcore smokers smoked more than ambivalent non-hardcore smokers, *p* = .028. and more than disengaged non-hardcore smokers, *p* < .001. Ambivalent non-hardcore smokers smoked more than disengaged non-hardcore smokers, *p* = .002.

#### Quitting self-efficacy

Quitting self-efficacy differed between profiles, *F*(2,334) = 10.844, *p* < .001, η^2^ = .061. Post-hoc analyses revealed that *resistant* non-hardcore smokers had more quitting self-efficacy than both receptive non-hardcore smokers, *p* < .001. and ambivalent non-hardcore smokers, *p* < .001. We found no difference between receptive and ambivalent non-hardcore smokers, *p* = .421.

## Discussion

In this study we used the perceived pros and cons of smoking and quitting to identify profiles in both hardcore smokers and non-hardcore smokers. We found three profiles in hardcore smokers and three in non-hardcore smokers.

Our findings supported our hypotheses about the composition of profiles in hardcore smokers. Dijkstra and De Vries [12] distinguished between motivated, unmotivated and disengaged smokers. In line with Dijkstra and De Vries [12], we found one profile whose members were receptive to quitting (i.e. agreed with the cons of smoking and the pros of quitting); one profile whose members were ambivalent towards quitting (i.e. scored about neutral on all four pros and cons scales); and one profile whose members were resistant to quitting (i.e. disagreed with the cons of smoking and the pros of quitting). We labelled members of these profiles ‘receptive, ‘ambivalent’ and ‘*resistant*’.

Our results further suggested that the differences in perceived pros and cons between profiles in hardcore smokers could be partially explained by nicotine dependence. Receptive hardcore smokers, who had a more positive view on quitting than ambivalent hardcore smokers, were also more nicotine dependent than ambivalent hardcore smokers. This contradiction could be explained by the association between nicotine dependence and poorer health status [33]. Receptive hardcore smokers may have faced health-related problems (e.g. coughing) and other smoking-related issues (e.g. smoking restrictions) more frequently than ambivalent hardcore smokers. They may have been more aware of the negative consequences of smoking and thus more positive towards quitting.

Non-hardcore smokers showed a different pattern of profiles than hardcore smokers. In both samples the first two profiles were ‘receptive’ and ‘ambivalent’. Whereas the third profile was ‘*resistant*’ in hardcore smokers, it was ‘disengaged’ in non-hardcore smokers. The former was rather negative about quitting, while the latter appeared to be uninvolved in either smoking or quitting.

Differences between these profiles might be explained by both daily tobacco consumption and nicotine dependence. Disengaged non-hardcore smokers had lower tobacco consumption and nicotine dependence than all other non-hardcore smokers. Having a low tobacco consumption could make quitting less urgent for these disengaged smokers than for other smokers. *Resistant* hardcore smokers, on the other hand, had a high nicotine dependence, but perceived very few cons of smoking. Perhaps they experienced high levels of cognitive dissonance, because they also believed (or at least pretend) that smoking has many benefits and that quitting has few [34,35]. Tobacco consumption may thus explain the difference between *resistant* hardcore smokers and disengaged non-hardcore smokers. Its role is different between hardcore and non-hardcore smokers, which in line with the literature, that states that both groups of smokers are distinct [5].

We found several differences between hardcore and non-hardcore smokers. As said, we found that hardcore smokers also smoked more cigarettes per day and had smoked more years in their lives. These differences are explained by the way the two groups were defined. As nicotine dependence is strongly related to cigarettes per day, it is not surprising that hardcore smokers scored higher on nicotine dependence as well. However, we also found other differences. In line with previous research, quitting self-efficacy was lower among hardcore smokers than among non-hardcore smokers [6] and they had started smoking at a younger age. Hardcore smokers also saw more pros of smoking and cons of quitting. Since hardcore smokers are more nicotine dependent than non-hardcore smokers, quitting may be especially difficult for them. This could explain why hardcore smokers had lower quitting self-efficacy and lower quit intentions than non-hardcore smokers. It also explains why hardcore smokers saw more benefits of smoking and cost of quitting than non-hardcore smokers.

### Tobacco control strategies

Current tobacco-control strategies may not be sufficient to involve hardcore smokers in tobacco control [5,6]. The different profiles we found could help to develop individualized health messages or tailored interventions for this group.

While receptive hardcore smokers were more nicotine dependent, they were clearly aware of the disadvantages of smoking and the benefits of quitting. For members of this profile, there is no need to convince them that quitting smoking would be beneficial—they know that already. Instead, interventions targeting this group should aim to increase quitting self-efficacy or minimize nicotine dependence symptoms. Such interventions could stimulate the use of prescription medications and nicotine-replacement therapies. Pharmacotherapies—such as Verenicline and Bupropion—and nicotine-replacements therapies—such as nicotine gums or patches—are effective methods for quitting smoking [36,37].

Ambivalent hardcore smokers were less nicotine dependent that other hardcore smokers, but they showed ambivalence towards smoking and quitting. Perhaps they have never explicitly considered the advantages of quitting. Ambivalent hardcore smokers may therefore benefit from interventions incorporating motivational interviewing [38], in which participants are stimulated to explicitly discuss the pros and cons of behavioural change in an open and positive manner [39].

*Resistant* hardcore smokers may require more elaborate cognitive interventions. They may also benefit from motivational interviewing to target their pros and cons in the long term. Interventions targeting *resistant* hardcore smokers may need to be longer than those for ambivalent hardcore smokers, as longer motivational interviewing sessions have been shown to increase intervention effectiveness [38]. Resistant hardcore smokers may be unwilling to pursue treatment for tobacco addiction themselves. However, health care providers may propose such interventions during health care visits. Such health care visits may serve as a teachable moment and may stimulate resistant hardcore smokers to start an intervention to quit smoking [40]. To reduce the harm done by current smoking in the short term, interventions for *resistant* hardcore smokers could focus on smoking reduction. Smoking reduction is an effective strategy to quit smoking [41], especially when combined with nicotine replacement therapies [42].

### Strengths, limitations and future research

A major strength of this study is that we used two separate samples to compare profiles in hardcore and non-hardcore smokers. This allowed us to identify hardcore smokers as a distinct subgroup of smokers, that requires special attention in tobacco control. Another strength of our study is our use of elaborate sets of perceived pros and cons from previous focus group interviews among hardcore smokers. These pros and cons covered the full spectrum of perceived pros and cons relevant to hardcore smokers.

A possible limitation of our research is the use of online data collection. Although 94 percent of Dutch households have access to the internet [43], not all smokers aged 35–65 are willing to take part in an online panel. Since we collected data among online panel members, the results may not be completely generalizable to all hardcore smokers. Another limitation is the use of cross-sectional data. We have no data on the degree to which profile compositions vary over time. Future longitudinal research may help to identify variables that influence such possible variations. This could help to predict—and perhaps influence—perceived pros and cons in hardcore smokers.

In our study, both hardcore and non-hardcore smokers did not intend to quit within six months. Future research might also investigate profiles in non-hardcore smokers who are more willing to quit smoking. As such smokers are more distinct from hardcore smokers than the non-hardcore smokers in our study, their profiles may offer additional insight into the unique characteristics of hardcore smokers.

## Conclusions

We found three distinct profiles among hardcore smokers and each profile might require a different tobacco control approach. We also found that hardcore smokers started smoking earlier in life and have less quitting self-efficacy than non-hardcore smokers. They were also more nicotine dependent, had lower intention to quit smoking, and saw more pros of smoking and cons of quitting. Future research may help to develop theories and interventions for this group. Our study showed that many hardcore smokers are rather positive about quitting. If given the most appropriate intervention, they could thus be stimulated to quit smoking.
